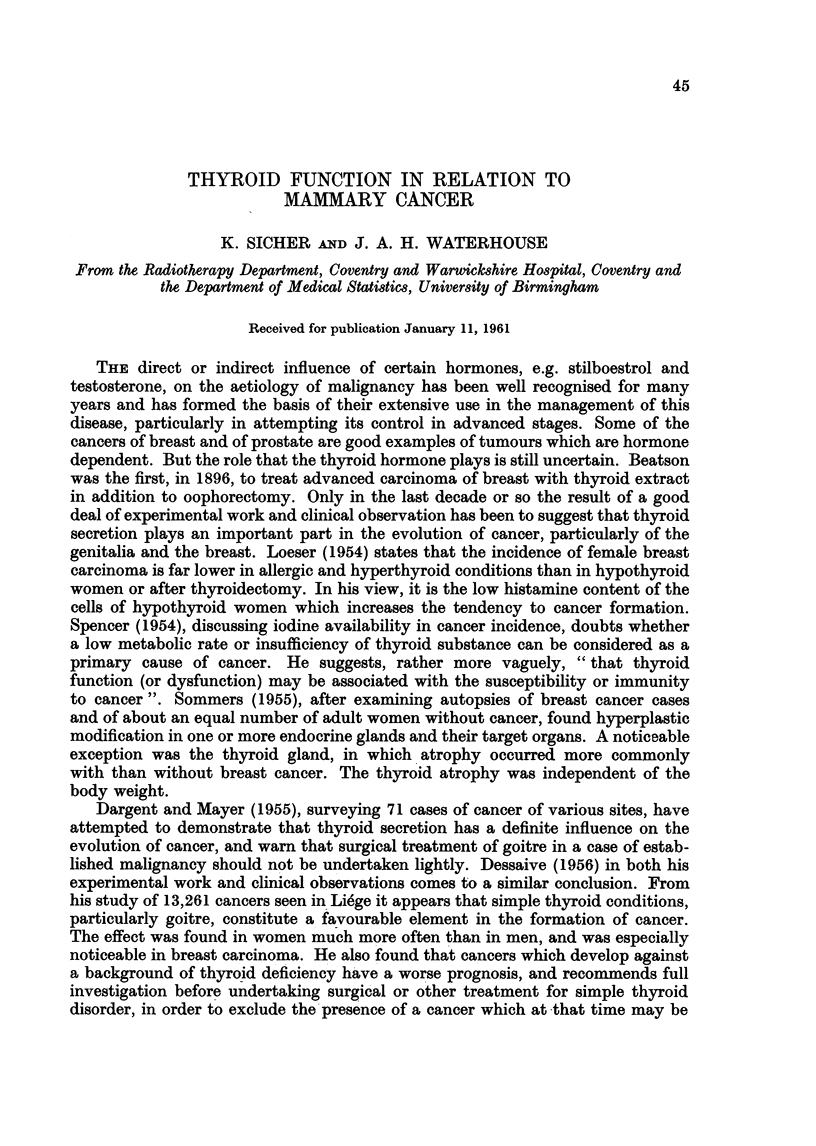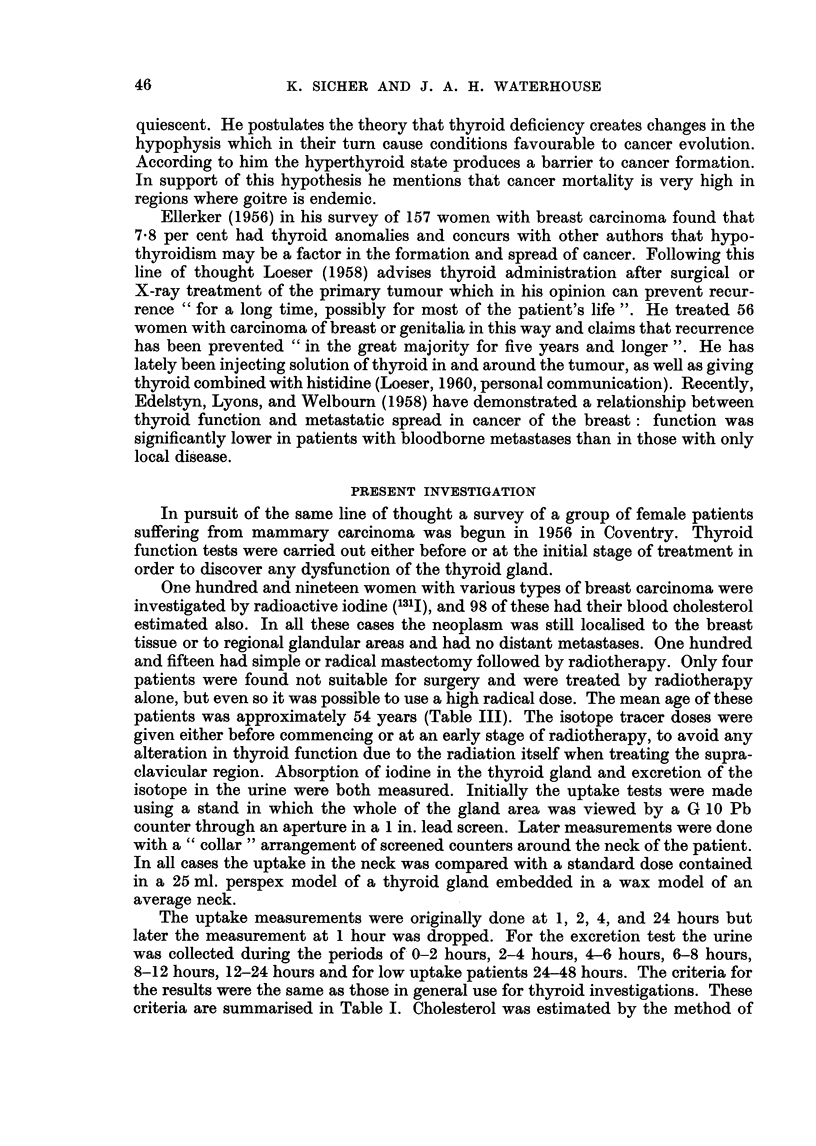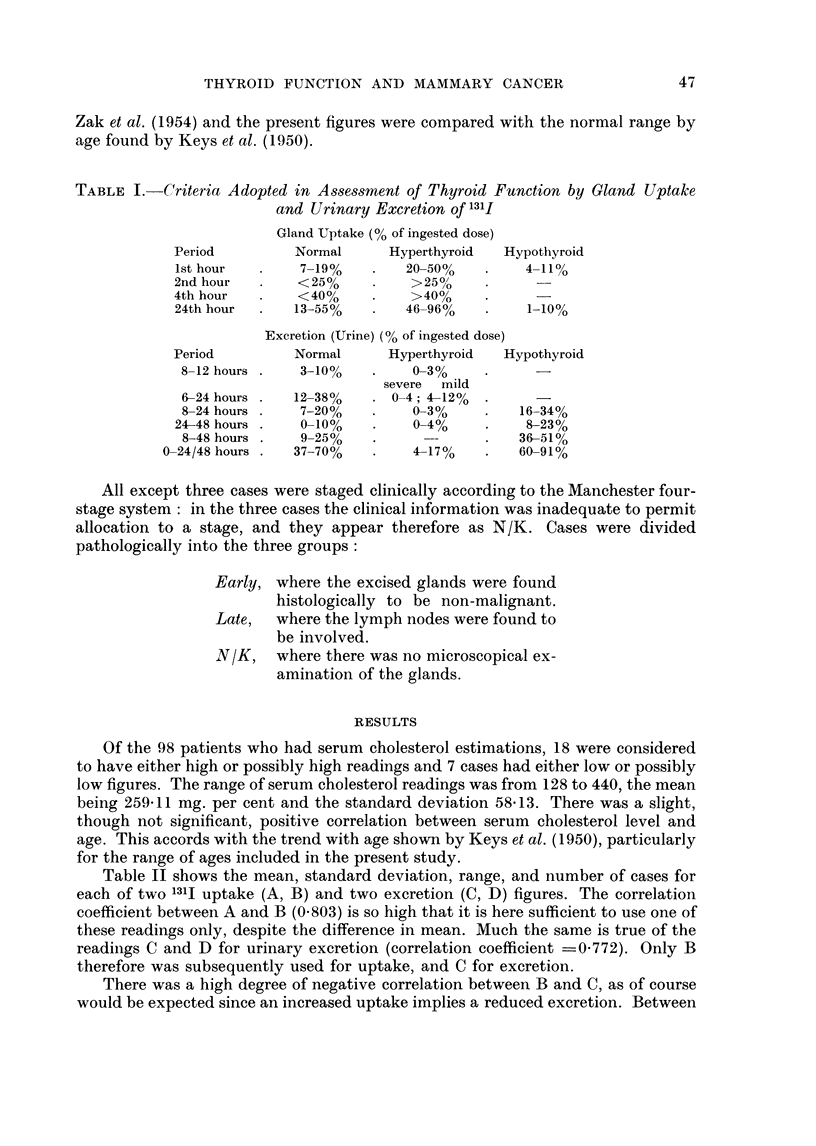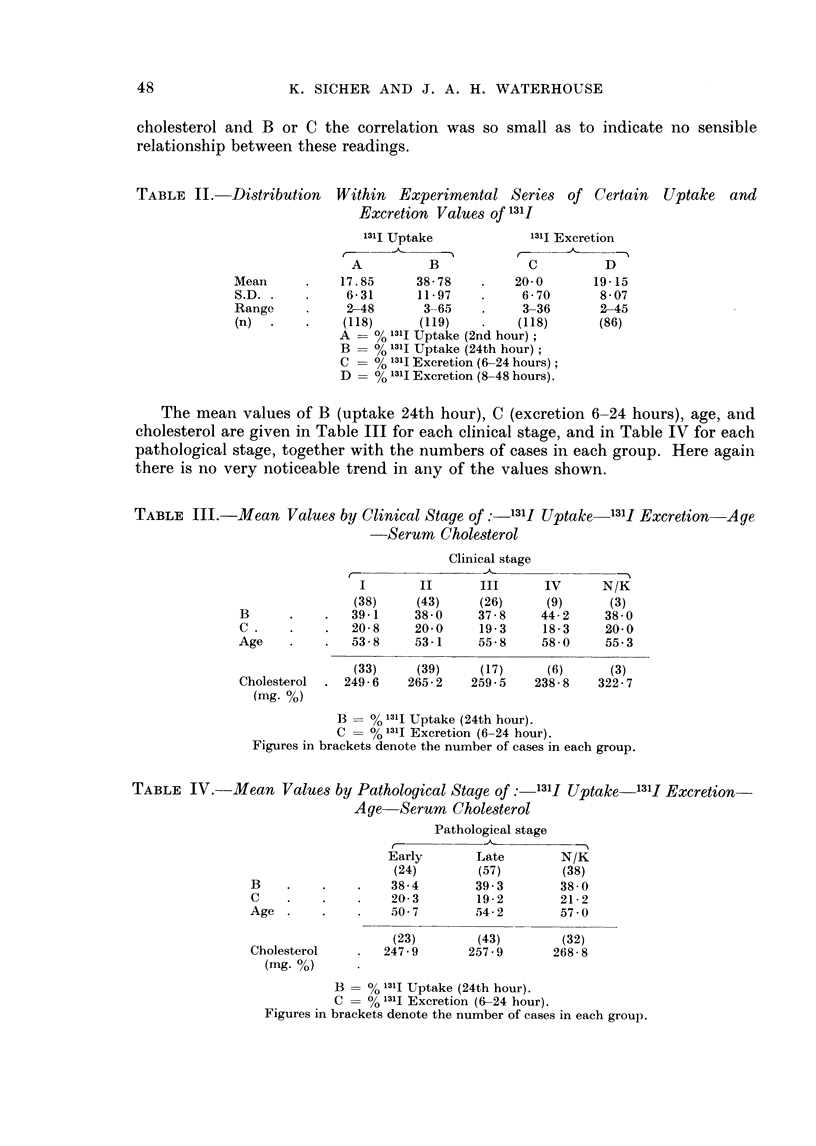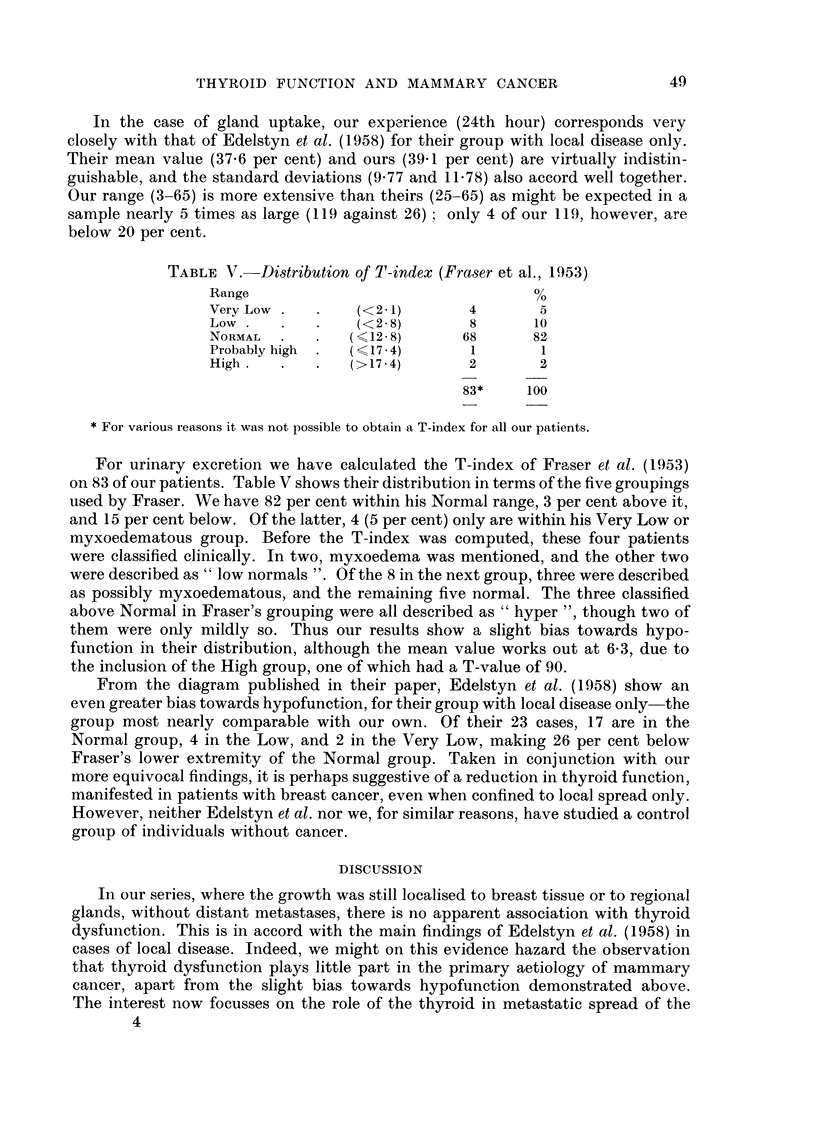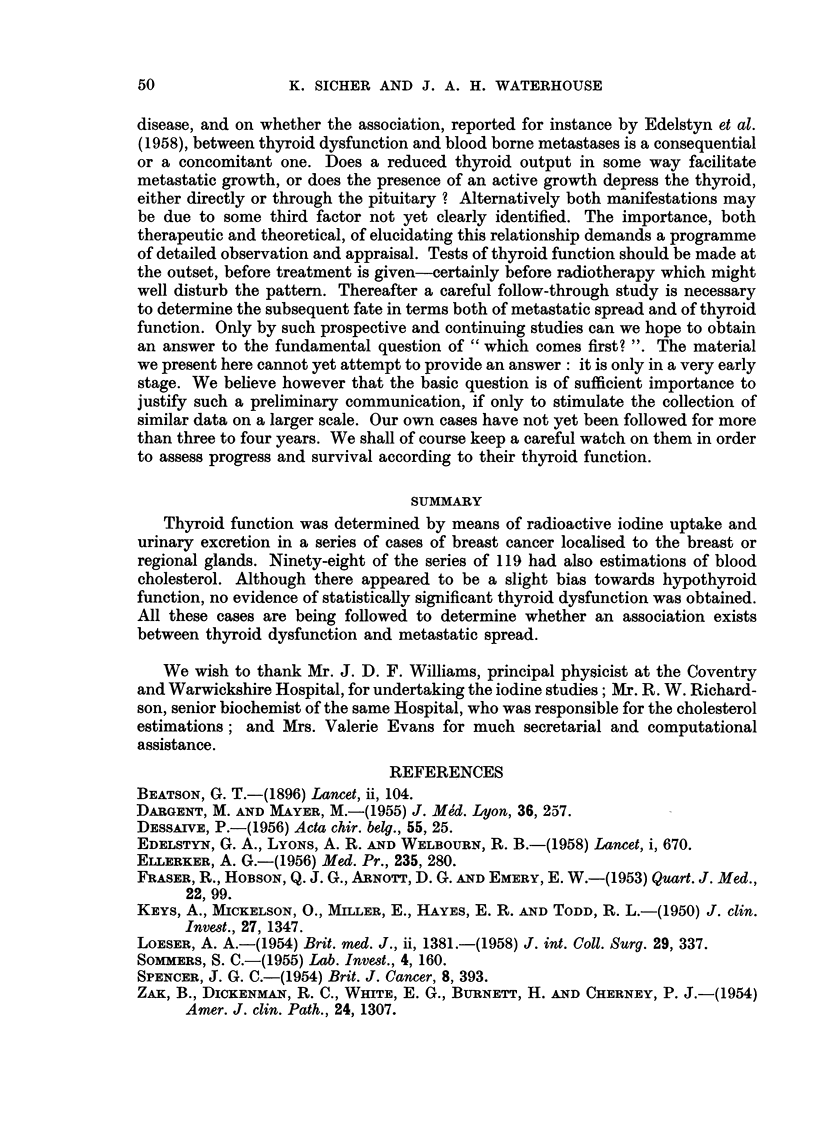# Thyroid Function in Relation to Mammary Cancer

**DOI:** 10.1038/bjc.1961.6

**Published:** 1961-03

**Authors:** K. Sicher, J. A. H. Waterhouse


### THYROID FUNCTION IN RELATION TO MAMMARY CANCER

K. SICHER AND J. A. H. WATERHOUSE

*From the Radiotherapy Department, Coventry and Warwickshire Hospital, Coventry and the Department of Medical Statistics, University of Birmingham*

Received for publication January 11, 1961

THE direct or indirect influence of certain hormones, e.g. stilboestrol and testosterone, on the aetiology of malignancy has been well recognised for many years and has formed the basis of their extensive use in the management of this disease, particularly in attempting its control in advanced stages. Some of the cancers of breast and of prostate are good examples of tumours which are hormone dependent. But the role that the thyroid hormone plays is still uncertain. Beatson was the first, in 1896, to treat advanced carcinoma of breast with thyroid extract in addition to oophorectomy. Only in the last decade or so the result of a good deal of experimental work and clinical observation has been to suggest that thyroid secretion plays an important part in the evolution of cancer, particularly of the genitalia and the breast. Loeser (1954) states that the incidence of female breast carcinoma is far lower in allergic and hyperthyroid conditions than in hypothyroid women or after thyroidectomy. In his view, it is the low histamine content of the cells of hypothyroid women which increases the tendency to cancer formation. Spencer (1954), discussing iodine availability in cancer incidence, doubts whether a low metabolic rate or insufficiency of thyroid substance can be considered as a primary cause of cancer. He suggests, rather more vaguely, " that thyroid function (or dysfunction) may be associated with the susceptibility or immunity to cancer ". Sommers (1955), after examining autopsies of breast cancer cases and of about an equal number of adult women without cancer, found hyperplastic modification in one or more endocrine glands and their target organs. A noticeable exception was the thyroid gland, in which atrophy occurred more commonly with than without breast cancer. The thyroid atrophy was independent of the body weight.

Dargent and Mayer (1955), surveying 71 cases of cancer of various sites, have attempted to demonstrate that thyroid secretion has a definite influence on the evolution of cancer, and warn that surgical treatment of goitre in a case of established malignancy should not be undertaken lightly. Dessaive (1956) in both his experimental work and clinical observations comes to a similar conclusion. From his study of 13,261 cancers seen in Liége it appears that simple thyroid conditions, particularly goitre, constitute a favourable element in the formation of cancer. The effect was found in women much more often than in men, and was especially noticeable in breast carcinoma. He also found that cancers which develop against a background of thyroid deficiency have a worse prognosis, and recommends full investigation before undertaking surgical or other treatment for simple thyroid disorder, in order to exclude the presence of a cancer which at that time may be quiescent. He postulates the theory that thyroid deficiency creates changes in the hypophysis which in their turn cause conditions favourable to cancer evolution. According to him the hyperthyroid state produces a barrier to cancer formation. In support of this hypothesis he mentions that cancer mortality is very high in regions where goitre is endemic.

Ellerker (1956) in his survey of 157 women with breast carcinoma found that 7·8 per cent had thyroid anomalies and concurs with other authors that hypothyroidism may be a factor in the formation and spread of cancer. Following this line of thought Loeser (1958) advises thyroid administration after surgical or X-ray treatment of the primary tumour which in his opinion can prevent recurrence " for a long time, possibly for most of the patient's life ". He treated 56 women with carcinoma of breast or genitalia in this way and claims that recurrence has been prevented " in the great majority for five years and longer ". He has lately been injecting solution of thyroid in and around the tumour, as well as giving thyroid combined with histidine (Loeser, 1960, personal communication). Recently, Edelstyn, Lyons, and Welbourn (1958) have demonstrated a relationship between thyroid function and metastatic spread in cancer of the breast: function was significantly lower in patients with bloodborne metastases than in those with only local disease.

### PRESENT INVESTIGATION

In pursuit of the same line of thought a survey of a group of female patients suffering from mammary carcinoma was begun in 1956 in Coventry. Thyroid function tests were carried out either before or at the initial stage of treatment in order to discover any dysfunction of the thyroid gland.

One hundred and nineteen women with various types of breast carcinoma were investigated by radioactive iodine ($^{131}I$), and 98 of these had their blood cholesterol estimated also. In all these cases the neoplasm was still localised to the breast tissue or to regional glandular areas and had no distant metastases. One hundred and fifteen had simple or radical mastectomy followed by radiotherapy. Only four patients were found not suitable for surgery and were treated by radiotherapy alone, but even so it was possible to use a high radical dose. The mean age of these patients was approximately 54 years (Table III). The isotope tracer doses were given either before commencing or at an early stage of radiotherapy, to avoid any alteration in thyroid function due to the radiation itself when treating the supraclavicular region. Absorption of iodine in the thyroid gland and excretion of the isotope in the urine were both measured. Initially the uptake tests were made using a stand in which the whole of the gland area was viewed by a G 10 Pb counter through an aperture in a 1 in. lead screen. Later measurements were done with a " collar " arrangement of screened counters around the neck of the patient. In all cases the uptake in the neck was compared with a standard dose contained in a 25 ml. perspex model of a thyroid gland embedded in a wax model of an average neck.

The uptake measurements were originally done at 1, 2, 4, and 24 hours but later the measurement at 1 hour was dropped. For the excretion test the urine was collected during the periods of 0–2 hours, 2–4 hours, 4–6 hours, 6–8 hours, 8–12 hours, 12–24 hours and for low uptake patients 24–48 hours. The criteria for the results were the same as those in general use for thyroid investigations. These criteria are summarised in Table I. Cholesterol was estimated by the method of

Zak *et al.* (1954) and the present figures were compared with the normal range by age found by Keys *et al.* (1950).

TABLE I.—*Criteria Adopted in Assessment of Thyroid Function by Gland Uptake and Urinary Excretion of* $^{131}I$

Gland Uptake (% of ingested dose)

| Period | Normal | Hyperthyroid | Hypothyroid |
|---|---|---|---|
| 1st hour | 7–19% | 20–50% | 4–11% |
| 2nd hour | <25% | >25% | — |
| 4th hour | <40% | >40% | — |
| 24th hour | 13–55% | 46–96% | 1–10% |

Excretion (Urine) (% of ingested dose)

| Period | Normal | Hyperthyroid | Hypothyroid |
|---|---|---|---|
| 8–12 hours | 3–10% | 0–3% | — |
| | | severe mild | |
| 6–24 hours | 12–38% | 0–4 ; 4–12% | — |
| 8–24 hours | 7–20% | 0–3% | 16–34% |
| 24–48 hours | 0–10% | 0–4% | 8–23% |
| 8–48 hours | 9–25% | — | 36–51% |
| 0–24/48 hours | 37–70% | 4–17% | 60–91% |

All except three cases were staged clinically according to the Manchester four-stage system : in the three cases the clinical information was inadequate to permit allocation to a stage, and they appear therefore as N/K. Cases were divided pathologically into the three groups :

*Early*, where the excised glands were found histologically to be non-malignant.

*Late*, where the lymph nodes were found to be involved.

*N/K*, where there was no microscopical examination of the glands.

### RESULTS

Of the 98 patients who had serum cholesterol estimations, 18 were considered to have either high or possibly high readings and 7 cases had either low or possibly low figures. The range of serum cholesterol readings was from 128 to 440, the mean being 259·11 mg. per cent and the standard deviation 58·13. There was a slight, though not significant, positive correlation between serum cholesterol level and age. This accords with the trend with age shown by Keys *et al.* (1950), particularly for the range of ages included in the present study.

Table II shows the mean, standard deviation, range, and number of cases for each of two $^{131}$I uptake (A, B) and two excretion (C, D) figures. The correlation coefficient between A and B (0·803) is so high that it is here sufficient to use one of these readings only, despite the difference in mean. Much the same is true of the readings C and D for urinary excretion (correlation coefficient =0·772). Only B therefore was subsequently used for uptake, and C for excretion.

There was a high degree of negative correlation between B and C, as of course would be expected since an increased uptake implies a reduced excretion. Between cholesterol and B or C the correlation was so small as to indicate no sensible relationship between these readings.

TABLE II.—*Distribution Within Experimental Series of Certain Uptake and Excretion Values of* $^{131}I$

| | $^{131}$I Uptake | | $^{131}$I Excretion | |
|---|---|---|---|---|
| | A | B | C | D |
| Mean | 17.85 | 38·78 | 20·0 | 19·15 |
| S.D. | 6·31 | 11·97 | 6·70 | 8·07 |
| Range | 2–48 | 3–65 | 3–36 | 2–45 |
| (n) | (118) | (119) | (118) | (86) |

A = % $^{131}$I Uptake (2nd hour) ;

B = % $^{131}$I Uptake (24th hour) ;

C = % $^{131}$I Excretion (6–24 hours) ;

D = % $^{131}$I Excretion (8–48 hours).

The mean values of B (uptake 24th hour), C (excretion 6–24 hours), age, and cholesterol are given in Table III for each clinical stage, and in Table IV for each pathological stage, together with the numbers of cases in each group. Here again there is no very noticeable trend in any of the values shown.

TABLE III.—*Mean Values by Clinical Stage of :—*$^{131}I$ *Uptake—*$^{131}I$ *Excretion—Age —Serum Cholesterol*

| | Clinical stage | | | | |
|---|---|---|---|---|---|
| | I | II | III | IV | N/K |
| | (38) | (43) | (26) | (9) | (3) |
| B | 39·1 | 38·0 | 37·8 | 44·2 | 38·0 |
| C | 20·8 | 20·0 | 19·3 | 18·3 | 20·0 |
| Age | 53·8 | 53·1 | 55·8 | 58·0 | 55·3 |
| | (33) | (39) | (17) | (6) | (3) |
| Cholesterol (mg. %) | 249·6 | 265·2 | 259·5 | 238·8 | 322·7 |

B = % $^{131}$I Uptake (24th hour).
C = % $^{131}$I Excretion (6–24 hour).
Figures in brackets denote the number of cases in each group.

TABLE IV.—*Mean Values by Pathological Stage of :—*$^{131}I$ *Uptake—*$^{131}I$ *Excretion—Age—Serum Cholesterol*

| | Pathological stage | | |
|---|---|---|---|
| | Early | Late | N/K |
| | (24) | (57) | (38) |
| B | 38·4 | 39·3 | 38·0 |
| C | 20·3 | 19·2 | 21·2 |
| Age | 50·7 | 54·2 | 57·0 |
| | (23) | (43) | (32) |
| Cholesterol (mg. %) | 247·9 | 257·9 | 268·8 |

B = % $^{131}$I Uptake (24th hour).
C = % $^{131}$I Excretion (6–24 hour).
Figures in brackets denote the number of cases in each group.

In the case of gland uptake, our experience (24th hour) corresponds very closely with that of Edelstyn *et al.* (1958) for their group with local disease only. Their mean value (37·6 per cent) and ours (39·1 per cent) are virtually indistinguishable, and the standard deviations (9·77 and 11·78) also accord well together. Our range (3–65) is more extensive than theirs (25–65) as might be expected in a sample nearly 5 times as large (119 against 26); only 4 of our 119, however, are below 20 per cent.

TABLE V.—*Distribution of T-index* (*Fraser* et al., 1953)

| Range | | | % |
|---|---|---|---|
| Very Low . . | ($<2·1$) | 4 | 5 |
| Low . . . | ($<2·8$) | 8 | 10 |
| NORMAL . . | ($\leqslant 12·8$) | 68 | 82 |
| Probably high . | ($\leqslant 17·4$) | 1 | 1 |
| High . . . | ($>17·4$) | 2 | 2 |
| | | 83* | 100 |

* For various reasons it was not possible to obtain a T-index for all our patients.

For urinary excretion we have calculated the T-index of Fraser *et al.* (1953) on 83 of our patients. Table V shows their distribution in terms of the five groupings used by Fraser. We have 82 per cent within his Normal range, 3 per cent above it, and 15 per cent below. Of the latter, 4 (5 per cent) only are within his Very Low or myxoedematous group. Before the T-index was computed, these four patients were classified clinically. In two, myxoedema was mentioned, and the other two were described as " low normals ". Of the 8 in the next group, three were described as possibly myxoedematous, and the remaining five normal. The three classified above Normal in Fraser's grouping were all described as " hyper ", though two of them were only mildly so. Thus our results show a slight bias towards hypofunction in their distribution, although the mean value works out at 6·3, due to the inclusion of the High group, one of which had a T-value of 90.

From the diagram published in their paper, Edelstyn *et al.* (1958) show an even greater bias towards hypofunction, for their group with local disease only—the group most nearly comparable with our own. Of their 23 cases, 17 are in the Normal group, 4 in the Low, and 2 in the Very Low, making 26 per cent below Fraser's lower extremity of the Normal group. Taken in conjunction with our more equivocal findings, it is perhaps suggestive of a reduction in thyroid function, manifested in patients with breast cancer, even when confined to local spread only. However, neither Edelstyn *et al.* nor we, for similar reasons, have studied a control group of individuals without cancer.

### DISCUSSION

In our series, where the growth was still localised to breast tissue or to regional glands, without distant metastases, there is no apparent association with thyroid dysfunction. This is in accord with the main findings of Edelstyn *et al.* (1958) in cases of local disease. Indeed, we might on this evidence hazard the observation that thyroid dysfunction plays little part in the primary aetiology of mammary cancer, apart from the slight bias towards hypofunction demonstrated above. The interest now focusses on the role of the thyroid in metastatic spread of the disease, and on whether the association, reported for instance by Edelstyn *et al.* (1958), between thyroid dysfunction and blood borne metastases is a consequential or a concomitant one. Does a reduced thyroid output in some way facilitate metastatic growth, or does the presence of an active growth depress the thyroid, either directly or through the pituitary ? Alternatively both manifestations may be due to some third factor not yet clearly identified. The importance, both therapeutic and theoretical, of elucidating this relationship demands a programme of detailed observation and appraisal. Tests of thyroid function should be made at the outset, before treatment is given—certainly before radiotherapy which might well disturb the pattern. Thereafter a careful follow-through study is necessary to determine the subsequent fate in terms both of metastatic spread and of thyroid function. Only by such prospective and continuing studies can we hope to obtain an answer to the fundamental question of " which comes first? ". The material we present here cannot yet attempt to provide an answer : it is only in a very early stage. We believe however that the basic question is of sufficient importance to justify such a preliminary communication, if only to stimulate the collection of similar data on a larger scale. Our own cases have not yet been followed for more than three to four years. We shall of course keep a careful watch on them in order to assess progress and survival according to their thyroid function.

SUMMARY

Thyroid function was determined by means of radioactive iodine uptake and urinary excretion in a series of cases of breast cancer localised to the breast or regional glands. Ninety-eight of the series of 119 had also estimations of blood cholesterol. Although there appeared to be a slight bias towards hypothyroid function, no evidence of statistically significant thyroid dysfunction was obtained. All these cases are being followed to determine whether an association exists between thyroid dysfunction and metastatic spread.

We wish to thank Mr. J. D. F. Williams, principal physicist at the Coventry and Warwickshire Hospital, for undertaking the iodine studies ; Mr. R. W. Richardson, senior biochemist of the same Hospital, who was responsible for the cholesterol estimations ; and Mrs. Valerie Evans for much secretarial and computational assistance.

REFERENCES

Beatson, G. T.—(1896) *Lancet*, ii, 104.
Dargent, M. and Mayer, M.—(1955) *J. Méd. Lyon*, **36**, 257.
Dessaive, P.—(1956) *Acta chir. belg.*, **55**, 25.
Edelstyn, G. A., Lyons, A. R. and Welbourn, R. B.—(1958) *Lancet*, i, 670.
Ellerker, A. G.—(1956) *Med. Pr.*, **235**, 280.
Fraser, R., Hobson, Q. J. G., Arnott, D. G. and Emery, E. W.—(1953) *Quart. J. Med.*, **22**, 99.
Keys, A., Mickelson, O., Miller, E., Hayes, E. R. and Todd, R. L.—(1950) *J. clin. Invest.*, **27**, 1347.
Loeser, A. A.—(1954) *Brit. med. J.*, ii, 1381.—(1958) *J. int. Coll. Surg.* **29**, 337.
Sommers, S. C.—(1955) *Lab. Invest.*, **4**, 160.
Spencer, J. G. C.—(1954) *Brit. J. Cancer*, **8**, 393.
Zak, B., Dickenman, R. C., White, E. G., Burnett, H. and Cherney, P. J.—(1954) *Amer. J. clin. Path.*, **24**, 1307.